# Functional Duality of Chondrocyte Hypertrophy and Biomedical Application Trends in Osteoarthritis

**DOI:** 10.3390/pharmaceutics13081139

**Published:** 2021-07-26

**Authors:** Sunghyun Park, Alvin Bello, Yoshie Arai, Jinsung Ahn, Dohyun Kim, Kyung-Yup Cha, Inho Baek, Hansoo Park, Soo-Hong Lee

**Affiliations:** 1 Department of Medical Biotechnology, Dongguk University-Seoul, Seoul 04620, Korea; nightsky9836@naver.com (S.P.); abacerobello@gmail.com (A.B.); ruddov@gmail.com (Y.A.); jinsung0711@gmail.com (J.A.); ehgus1006@hanmail.net (D.K.); cha88990@naver.com (K.-Y.C.); dlsghdlscks@naver.com (I.B.); 2 Department of Biomedical Science, CHA University, 335 Pangyo-ro, Bundang-gu, Seongnam-si 13488, Korea; 3 School of Integrative Engineering, Chung-ang University, 84 Heukseok-ro, Dongjak-gu, Seoul 06974, Korea; heyshoo@gmail.com

**Keywords:** chondrocyte hypertrophy, osteoarthritis, exosome, 3D scaffolds, small molecules

## Abstract

Chondrocyte hypertrophy is one of the key indicators in the progression of osteoarthritis (OA). However, compared with other OA indications, such as cartilage collapse, sclerosis, inflammation, and protease activation, the mechanisms by which chondrocyte hypertrophy contributes to OA remain elusive. As the pathological processes in the OA cartilage microenvironment, such as the alterations in the extracellular matrix, are initiated and dictated by the physiological state of the chondrocytes, in-depth knowledge of chondrocyte hypertrophy is necessary to enhance our understanding of the disease pathology and develop therapeutic agents. Chondrocyte hypertrophy is a factor that induces OA progression; it is also a crucial factor in the endochondral ossification. This review elaborates on this dual functionality of chondrocyte hypertrophy in OA progression and endochondral ossification through a description of the characteristics of various genes and signaling, their mechanism, and their distinguishable physiological effects. Chondrocyte hypertrophy in OA progression leads to a decrease in chondrogenic genes and destruction of cartilage tissue. However, in endochondral ossification, it represents an intermediate stage at the process of differentiation of chondrocytes into osteogenic cells. In addition, this review describes the current therapeutic strategies and their mechanisms, involving genes, proteins, cytokines, small molecules, three-dimensional environments, or exosomes, against the OA induced by chondrocyte hypertrophy. Finally, this review proposes that the contrasting roles of chondrocyte hypertrophy are essential for both OA progression and endochondral ossification, and that this cellular process may be targeted to develop OA therapeutics.

## 1. Introduction

Osteoarthritis (OA) is one of the most common chronic diseases worldwide. Globally, over 300 million people suffer from OA, which is accompanied by constant pain and cartilage degeneration [1]. Different ratios of occurrence are shown, according to sex, race, age, and job. However, OA is a disease that typically occurs in people over 55 years of age, regardless of those factors [2].

Meanwhile, OA is the result of a pathological and clinical range of disorders caused by the functional and structural failure of synovial joints. Moreover, the chronic pain of OA causes constant psychological stress and physical disability [3]. Therefore, overcoming this disease is important for well-being in an aging modern society. Although any joint is susceptible to OA, the most commonly affected ones are the wrist, elbow, spine, knee, and ankle joints. The disease pathology of OA has been studied and is based on various scientific or clinical observations. OA symptoms primarily include cartilage collapse, sclerosis, synovitis, inflammation, osteophyte formation, and protease activation. Current treatments provide only symptomatic relief, as they do not target the fundamental cause of OA. Furthermore, end-stage OA can only be treated via replacement surgery, which is an expensive and risky procedure [4]. Therefore, the early detection and treatment of OA is necessary to prevent disease progression.

Chondrocytes are the most abundant cell type in the cartilage tissue and have various functions in maintaining healthy cartilage. For example, healthy chondrocytes secrete various type of extracellular matrix (ECM) components, such as collagen, proteoglycans, hyaluronan, and glycoproteins [5]. However, as in any other cell, chondrocytes degenerate due to aging, excessive mechanical load, or disease complications, such as abnormal metabolism and autoimmune disorders [6]. Consequently, chondrocytes lose their functionality and secrete fibrous ECM components, such as collagen type I (COL1) and X (COL10), as well as ECM-degrading proteases, such as matrix metalloproteinase-13 (MMP13), disintegrin, and metalloproteinase with thrombospondin motifs 5 (ADAMTS5), all of which promote OA.

The chondrocyte degeneration during OA concurs with chondrocyte hypertrophy. Thus, it appears that chondrocyte hypertrophy is correlated with OA progression. However, chondrocyte hypertrophy is not always harmful; in fact, it is a highly essential process in endochondral ossification during bone development. For instance, studies on endochondral ossification have reported that hypertrophic chondrocytes contribute to bone formation by differentiating into osteoblasts and osteocytes [7,8]. This review elucidates the dual functionality of chondrocyte hypertrophy in OA progression and endochondral ossification. Further, this review describes the recent trends in the treatment of OA related to chondrocyte hypertrophy, including genetic intervention and the application of biomolecules, chemical compounds, three-dimensional (3D) microenvironments, or exosomes.

## 2. Dual Function of Chondrocyte Hypertrophy

### 2.1. Chondrocyte Hypertrophy in Endochondral Ossification

Bone development involves two different processes—intramembranous ossification and endochondral ossification. Mesenchymal stem cells (MSCs) can directly differentiate into bone cells, including osteoblasts, via intramembranous ossification. This process is involved in the formation of flat bones, such as the skull, mandible, and clavicles [9]. Alternatively, MSCs can differentiate into chondrocyte precursors, which subsequently provide a substrate for further bone morphogenesis, a phenomenon called endochondral ossification. This process is responsible for the formation and elongation of long bones, as well as bone healing [10].

In endochondral ossification, the growth plate, which includes the cartilage tissue, is replaced and transformed into bone [11]. In this process, the cartilage tissue expands through chondrocyte proliferation, followed by chondrocyte hypertrophy, and finally undergoes ossification. Chondrocyte hypertrophy is primarily associated with terminal differentiation during endochondral ossification in the hypertrophic zone, as a physiological mechanism of skeletal development [12]. The growth plate is divided into three zones with respect to the endochondral ossification—the resting, proliferating, and hypertrophic zones. The chondrocytes in the resting zone secrete antiangiogenic factors and synthesize an ECM that mainly consists of collagen type II (COL2) and proteoglycans. In the proliferating zone, the flattened chondrocytes proliferate and align in columns. SRY-box transcription factor 9 (SOX9) acts as a major factor in the proliferating zone and binds to the cis element present in the COL2 α1 chain (COL2A1) gene, thereby stimulating the differentiation of MSCs into chondrocytes [13]. Finally, the chondrocytes in the hypertrophic zone are induced to undergo hypertrophy, ECM secretion, and matrix mineralization [14,15]. In addition, these chondrocytes secrete fibrotic ECM components, such as COL1 and COL10. Consequently, bone formation ensues [16,17].

Meanwhile, the chondrocytes in the hypertrophic zone are influenced by the ECM present in the cartilage, and by systemic and local soluble factors. The principal factors involved in the growth of cartilage and bone, as well as the chondrocyte hypertrophy, are insulin-like growth factor-1 (IGF-1), Wnt/β-catenin, runt-related transcription factor 2 (RUNX2), COL10, MMP13, transforming growth factor-beta (TGF-β) family members, bone morphogenetic protein (BMP), and Indian hedgehog (IHH) [18,19]. These factors induce the growth of cartilage and bone, as well as the hypertrophy in the endochondral ossification process used to form bones.

IGF-1 is secreted by the liver, an important organ that produces hormones that are essential for bone growth and development [20]. This growth factor induces chondrocyte proliferation and differentiation, via a mechanism involving the interaction of parathyroid hormone-related protein (PTHrP) with IHH. In addition to the liver, chondrocytes also express IGF-1 early during their development. Subsequently, IGF-1 regulates the PTHrP/IHH feedback loop, whereby PTHrP is upregulated. PTHrP induces cartilage cell proliferation; thus, cartilage thickness is increased. Hence, the stimulation of the interaction between PTHrP and IHH induces chondrocytes to undergo hypertrophy to form bone [21,22].

The canonical Wnt/β-catenin pathway is activated by the binding of the canonical Wnt ligands to a Frizzled-family receptor and an LRP5/6 co-receptor, causing an accumulation of β-catenin in the cytoplasm. Consequently, β-catenin translocates into the nucleus and activates the T-cell factor/lymphoid enhancer factor (TCF/LEF) transcription factors, which induce the transcription of Wnt target genes, such as RUNX2 [23].

Although the specific and definitive mechanism by which RUNX2 influences or induces hypertrophy is still not fully understood, studies suggest that RUNX2 binds to the promoters of COL10 and MMP13, activating their transcription and thereby stimulating chondrocyte hypertrophy [6]. COL10 is known to play an important role in the transformation of the cartilage into bone [24], and MMP13 degrades the ECM of the cartilage, and thus induces bone development [25,26]. Therefore, the transcriptional regulation of these factors by RUNX2 facilitates chondrocyte hypertrophy. Furthermore, RUNX2 also regulates the expression of various other factors associated with chondrocyte hypertrophy. For instance, MMP13 and RUNX2 are closely related in chondrocyte hypertrophy. In the late stages of hypertrophy, chondrocytes show similar MMP13 and RUNX2 expression patterns to those of osteoblasts. During bone formation, MMP13 transcription is controlled by RUNX2 [27]. Additionally, CCAAT/enhancer-binding protein β (C/EBPβ) also closely interacts with RUNX2. C/EBPβ, as the transcriptional factor of RUNX2 during cartilage development, is implicated in the pathogenesis of osteoarthritis. In this case, C/EBPβ and RUNX2 bind together to induce the MMP13 expression. In endochondral ossification, the MMP13 induces chondrocyte hypertrophy and hypertrophic chondrocytes into terminally differentiated chondrocytes. Subsequently, terminally differentiated chondrocytes form bones via the ossification process. Hence, the MMP13 also plays a role in pathogenic conditions such as osteoarthritis by degrading cartilage ECM and inducing the chondrocyte hypertrophy, and the pathogenic roles of MMP13 are elucidated in the next chapter, ‘Hypertrophy in cartilage diseases’ [28,29].

The TGF-β family members are also involved in the development of chondrocytes [30]. TGF-β members induce Smad 1/5/8-signaling through activin-like kinase, which results in RUNX2-mediated upregulation of hypertrophy-associated factors, including MMP13 and COL10 [31,32,33].

BMP-related signaling is one of the principal pathways regulating cartilage and bone development. Ablation of the type I BMP receptor (BMPR1A) gene in chondrocytes severely impairs the cartilage and bone structure, indicating that the BMP signaling acts at multiple levels during the chondrogenic differentiation of undifferentiated MSCs [34].

The PTHrP and IHH signaling pathways interact via a negative feedback loop. PTHrP upregulates SOX9 to induce chondrocyte proliferation and downregulates RUNX2, thus preventing chondrocyte hypertrophy. Moreover, it was previously determined that PTHrP inhibits the expression of IHH in pre-hypertrophic chondrocytes, preventing chondrogenic hypertrophy. However, when PTHrP was suppressed, chondrocyte underwent hypertrophy due to the expression of IHH [35,36]. Accordingly, the PTHrP/IHH negative feedback loop appears to be the main signaling pathway that regulates chondrocyte proliferation or hypertrophy [37].

**Figure 1:**
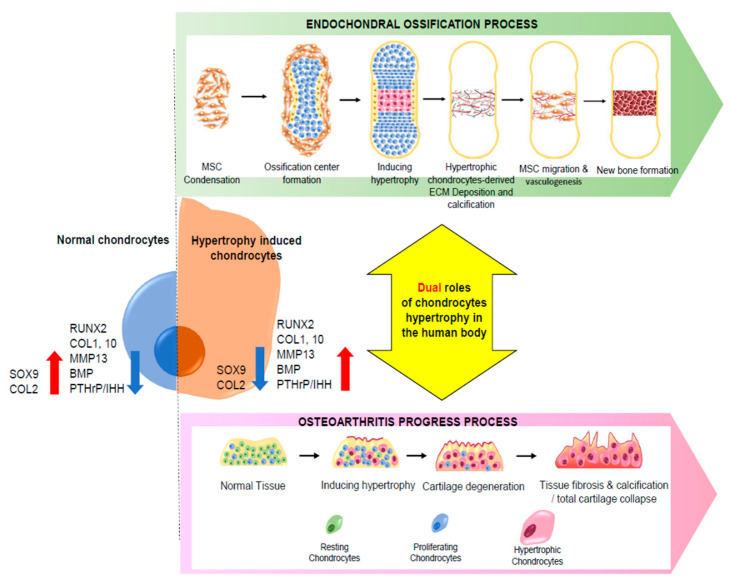
Schematic diagram of the dual role of chondrocyte hypertrophy in human physiology. Chondrocytes undergoing hypertrophy show different profiles of gene expression, ECM components, and secretome than those of undifferentiated chondrocytes. Hypertrophy-induced chondrocytes play different roles under different biological conditions.

Finally, chondrocytes undergo mineralization, an important process that occurs in the last phase of endochondral ossification. This mineralized matrix is eventually vascularized, enabling the infiltration of osteoblasts and osteoclasts [38]. Endochondral ossification has a significant effect on the proliferation and differentiation of chondrocytes, and chondrocyte hypertrophy is a crucial process in endochondral ossification (Figure 1).

Additionally, chondrocyte hypertrophy can also result from mechanical stress or a natural cause, such as aging or inflammation. In this case, chondrocyte hypertrophy plays different roles than in endochondral ossification. In the next section, the roles of chondrocyte hypertrophy in OA will be discussed.

### 2.2. Hypertrophy in Cartilage Diseases

Although chondrocyte hypertrophy plays an important role in endochondral ossification and cartilage-tissue maintenance, it can also have a negative impact on human physiology. Ectopic chondrocyte hypertrophy in the articular cartilage is known to be responsible for the pathogenesis of osteochondral diseases. The most common and representative osteochondral disease caused by chondrocyte hypertrophy is OA. In this disease, chondrocytes have a hypertrophic morphology, characterized by enlarged cells with an increased apoptotic rate. This phenotype is specifically attributed to the terminal stage of chondrocyte differentiation. Normally, terminal differentiation of the chondrocytes in the articular cartilage is suppressed. However, the articular cartilage in OA contains an excessive number of terminally differentiated chondrocytes compared with the healthy cartilage [39].

There are several known markers of chondrocyte hypertrophy, including MMP13 and COL10 [40,41], and several studies have reported that these markers are also expressed in OA mouse models [42,43]. It is important to know the different chondrocyte hypertrophy markers and their roles to understand the mechanism of chondrocyte hypertrophy in OA. Accordingly, in this subsection, the involvement of these markers in OA will be described.

OA gradually progresses through distinct stages and is regulated by various factors, such as secreted proteolytic enzymes and signaling pathways. During OA, hypertrophic chondrocytes secrete several proteolytic enzymes, such as ADAMTS and various MMPs that degrade the ECM, eventually leading to progressive cartilage degeneration, characterized by the erosion of the tissue surface, tissue softening, and fibrillation [44,45]. Matrix-degrading enzymes are deeply connected to the mechanical properties of the cartilage. For instance, the degradation of proteoglycans decreases the compressive stiffness of the tissue, and thus stimulates collagen denaturation [46]. Additionally, several factors regulating biomolecular processes are associated with chondrocyte hypertrophy in OA. RUNX2 is a representative transcription factor in chondrocyte hypertrophy. This transcription factor promotes the terminal differentiation of chondrocytes and upregulates COL10 [40,41].

In addition, C/EBPβ and hypoxia-inducible factor-2alpha (HIF-2α) were also reported to play important roles in chondrocyte terminal differentiation. HIF-2α upregulates COL10, MMP13 and VEGF. Moreover, HIF-2α has recently been reported as an inducer of C/EBPβ, a significant transcription factor that induces chondrocyte hypertrophy [47,48]. An increase in the expression of these factors causes fibrosis, collapse, and vascularization in the cartilage. In addition, many studies have proven a correlation between these regulators of ectopic chondrocyte hypertrophy and OA, using animal models. Several studies have demonstrated that the inhibition of various hypertrophy-promoting transcription factors, including RUNX2, C/EBPβ, and HIF-2α, protects against OA progression in mice [43,47,48,49]. Additionally, the partial loss of transcription factors related to chondrocyte hypertrophy, such as MMP13, delays the cartilage degeneration in OA mouse models [50]. Although various signaling pathways have been associated with chondrocyte hypertrophy, the Wnt/β-catenin and BMP/TGF-β pathways are the main regulatory pathways that induce hypertrophy, and these signaling pathways have been reported to be closely associated with OA [39,51]. One study reported that the Wnt/β-catenin signaling down-regulates NF-κβ and induces TGF-β/BMP signaling, consequently upregulating RUNX2, which upregulates COL10 and MMP13 [52]. As a result, these series of signaling processes ultimately lead to OA progression and chondrocyte hypertrophy.

Chondrocyte hypertrophy also plays a significant role in cartilage degeneration at the initiation stage of OA. It is well known that, during their terminal differentiation, chondrocytes synthesize COL10, while the cartilage matrix undergoes degradation [53]. A study reported that hypertrophic change in chondrocytes induces COL10 expression [41]. Additionally, terminally differentiated chondrocytes regulate the expression of ADAMTS, MMPs, and proteolytic enzymes, which degrade the ECM network [40]. In this stage, transcriptional regulators such as RUNX2 are activated, which, in turn, promotes the terminal differentiation of chondrocytes and upregulates COL10 [6]. Enzymes such as MMPs and aggrecanases initiate the degradation of collagen and proteoglycans in the ECM. Several studies have reported that cartilage collagen is degraded by MMP1 and MMP13, and cartilage proteoglycans are degraded by MMP3 and ADAMTS-4 [54,55]. Meanwhile, several studies have reported that repeated mechanical pressure on the articular cartilage induces MMP production and matrix collapse [56,57].

Chondrocyte hypertrophy is also involved in the advanced stages of OA. Once proteoglycans in the cartilage start to become degraded, the collagen in that region becomes weaker and eventually induces the chondrocytes to undergo hypertrophy by altering the cell microenvironment. The cartilage matrix undergoes degeneration and collapses due to the harsh microenvironment caused by these changes [6]. In addition, the RUNX2 activation is accelerated via a hypertrophic change in chondrocytes during the advanced stages of OA and induces the terminal differentiation of chondrocytes, which promotes the expression of COL10, alkaline phosphatase (ALP), and osteoprotegerin (OPG) [58,59]. Consequently, the matrix calcification is induced by the highly expressed alkaline phosphatase (ALP) and COL10 in this stage. ALP is involved in the ECM mineralization during the hypertrophic differentiation of chondrocytes and endochondral ossification [60,61,62]. Meanwhile, ALP is involved in ECM mineralization during the hypertrophic differentiation of chondrocytes and endochondral ossification [59,63,64,65,66]. Ultimately, hypertrophic changes in chondrocytes result in matrix calcification [59,67]. Indeed, the highly expressed OPG, a strong inhibitor of osteoclast differentiation and activation, prevents the progression of OA as a competitor for the receptor activator of nuclear factor kappa-Β ligand (RANKL). However, highly expressed OPG also cannot consistently prevent the OA from progressing due to the consistent external stress, or the physical and molecular breakdown of cartilage [62,68].

By the late stage of OA, the cartilage is already severely damaged. At this stage, the subchondral bone emerged to the surface of the cartilage inside the joint, and the cartilage tissue was crowded with heavily damaged, fissured, and fibrillated parts [69]. Moreover, focal bone cysts are observed because of the released cytokines, growth factors, and changed biomechanics [6] (Figure 1). Furthermore, OA is not the only disease resulting from chondrocyte hypertrophy. Recent studies have reported that, in degenerative intervertebral discs (IVDs), chondrocyte hypertrophic differentiation occurs, as in OA. These studies found that RUNX2, COL10, and ALP are upregulated in degenerative IVDs, in contrast with the low levels of these proteins that occur in non-degenerative IVDs [70,71,72]. In conclusion, as in the late stages of OA, chondrocyte hypertrophic differentiation induces IVD degeneration. Additionally, calcification in the late stages of IVD degeneration is affected by chondrocyte hypertrophic differentiation [60]. Since the progression of several osteochondral diseases is correlated with chondrocyte hypertrophy, inhibition of this cellular event could be effective in preventing or treating the progression of various osteochondral diseases. In the next chapter, the current approaches to treating OA induced by chondrocyte hypertrophy will be discussed.

## 3. Current Trends of the Treatment of Chondrocyte Hypertrophy-Induced OA

### 3.1. Genetic Interventions against Chondrocyte Hypertrophy

Since the underlying mechanism of chondrogenic hypertrophy is a rather complex process, several therapeutic strategies have been developed to target and inhibit the different key players and regulators of chondrocyte hypertrophy. The current strategies can be classified depending on the modalities used, such as genes, biomolecules, chemical inhibitors, and 3D biomaterials.

#### SOX 9

The transcription factor SOX9 is the master regulator of chondrogenic development [73,74] (Table 1). Several studies have suggested that SOX9 is highly expressed in all chondrogenic progenitors and is active in all stages of chondrogenic differentiation [75,76]. As one of the first genes expressed when MSCs are condensed during endochondral ossification, this transcription factor promotes the chondrogenic commitment of MSCs, ensures cellular survival and proliferation, inhibits hypertrophy, and functions as an upstream regulator of several genes involved in chondrogenic development [75,77]. The primary involvement of SOX9 in chondrogenesis has made it a promising candidate for gene-based treatment of chondrogenic hypertrophy. The adenoviral transduction of bone marrow mesenchymal stem cells (BM-MSCs) with SOX9 has been shown to induce them to undergo chondrogenesis and differentiate into nucleus pulposus cells in vitro [78]. In another study, it was found that the over-expression of SOX9 enhances chondrogenesis in BM-MSCs on both in-vitro and in-vivo set-ups [79].

It is also important to note that SOX9 activity might be enhanced via a combinatorial therapy including other SOX genes, such as *SOX5* an *SOX6*. The three proteins are known to simultaneously bind to the promoters of *COL2A* and *ACAN* and cooperatively activate the transcription of these genes, thereby promoting chondrogenic differentiation while inhibiting chondrogenic hypertrophy [80,81].

### 3.2. Histone Deacetylase 4 (HDAC4)

Another transcription factor that has been determined to regulate chondrocyte hypertrophy is HDAC4. Generally, HDACs remove acetyl moieties from the lysine residues of histone proteins, thereby inducing chromatin condensation and transcriptional repression of the affected genes [82,83]. HDACs are known epigenetic regulators of histone functions and gene expression [84]. There are several sub-classes of HDACs (I, IIa, IIb, III, and IV), depending on sequence homology, and HDAC4 falls under class IIa. HDAC4 plays a key role in several biological processes, such as cardiomyogenesis [83,85], neurogenesis [86], cancer progression [87], and cartilage and bone development [82,88].

In osteochondrogenesis, HDAC4 regulates chondrocyte hypertrophy by directly interacting with and inhibiting RUNX2 and myocyte-specific enhancer factor 2C (MEF2C). RUNX2 and MEF2 are known to promote osteogenesis by inducing hypertrophy in chondrocytes. The adenoviral transduction of HDAC in rat chondrocytes has been found to attenuate cartilage degeneration by inhibiting the expression of RUNX2, MMP13, and COL10. Moreover, overexpression of HDAC4 upregulates COL2 and ACAN, indicating a chondroprotective effect of HDAC4 [89]. In another study, HDAC4 overexpression was found to promote TGF-β1–induced chondrogenesis and inhibit cell hypertrophy [90].

#### NKX3.2

NKX3.2 is the human homolog of the fly bagpipe gene (*BAPX1*), which plays a key role in the development of the gut musculature [91]. However, in mammals, NKX3.2 is a known transcription factor that regulates osteochondral development [92]. Mutational experiments on NKX3.2/BAPX1 resulted in lethal skeletal dysplasia [93], asplenia, and abnormal formation of the axial skeleton and skull [94]. These observations suggest that NKX3.2 may also be a key regulator in chondrocyte maturation and hypertrophy. A study has shown that the retroviral transduction of NKX3.2 in pre-somitic mesoderm inhibits the expression of markers of chondrocyte maturation and hypertrophy, such as RUNX2 and COL10 [95]. The overexpression of NKX3.2 in C3H10T1/2 cells promotes chondrogenesis via binding to the enhancer segments of *COL2A1*, whereby the expression of *SOX9* is induced and chondrogenic differentiation is consequently enhanced [96].

Aside from these transcription factors, other candidate genes, such as E2F transcription factor 1 (*E2F1*) [97] and ZBTB20/ZNF288 [98], may be therapeutically targeted to inhibit cartilage hypertrophy and degeneration, thereby suppressing the progression of OA.

### 3.3. Protein Interventions for Chondrocyte Hypertrophy

#### 3.3.1. TGF-β1

TGF-β is a large family of growth factors that are known to regulate several biological processes in the body. TGF-β signaling has been known to direct embryonic development, and cellular proliferation and differentiation. This pathway is also involved in the progression of several human diseases, such as cancer and cardiovascular, reproductive, and musculo-skeletal diseases [39,99]. Although the TGF-β superfamily includes many members, such as activins, inhibins, and BMPs, there are three general prototypic isoforms of TGF-β—TGF-β1, TGF-β2, and TGF-β3 [100]. There is a considerable amount of evidence suggesting that all three isoforms are involved in chondrocyte and cartilage development, albeit to varying degrees. In a study on osteochondral ossification in the chick, it was found that the three isoforms are differentially expressed and localized during the different stages (proliferating, early hypertrophic, transitioning, and even hypertrophic chondrocytes), indicating that TGF-β is indeed a marker of chondrocyte differentiation [101].

Among the three studied isoforms, TGF-β1 has been widely accepted to prevent chondrocyte hypertrophy. In a study involving the in vitro differentiation of rat epiphysial chondrocytes, it was shown that exogenous TGF-β1 prevents the terminal differentiation of chondrocytes and reversibly prevents their hypertrophy [102]. In another study, TGF-β1 was considered as a protective regulator that inhibits chondrocyte hypertrophy by maintaining the production of GAG and other ECM components [103]. A similar stimulatory effect on chondrocytes was observed in another study, wherein TGF-β1 upregulated hyaluronan synthase and hyaluronic acid (HA) [104]. Due to the apparent role of TGF-β1 in chondrogenesis and hypertrophy, this cytokine was used as the primary growth factor in the conventional chondrogenic differentiation medium for MSCs.

The roles of TGF-β3 in the induction of chondrogenesis and inhibition of hypertrophy have also been assessed. In a 3D chondrogenic culture of human-induced pluripotent stem cells (iPSCs) in a polyethylene glycol (PEG) hydrogel, it was shown that TGF-β3 induces chondrogenesis and reduces hypertrophy by upregulating COL2 and ACAN [105]. In another study, the use of TGF-β3 in a pellet culture model of adipose-derived MSCs was shown to promote their chondrogenic differentiation and prevent their hypertrophy [106]. Indeed, the use of TGF-β growth factors is a potent approach to generate cartilage tissue and prevent chondrocyte hypertrophy. However, it is important to consider that TGF-β is also important for osteogenesis; therefore, proper dosing at each stage of chondrocyte development might be required to maximize the therapeutic effect of TGF-β.

#### 3.3.2. PTHrP

PTHrP was first discovered and characterized in 1987 as a primary factor that promotes malignant hypercalcemia [107,108]. PTHrP is a member of the classical sub-family of G protein-coupled receptors and is abundantly expressed in several locations, including the hair follicles, breast, and cartilage [35]. It is a 141 AA polypeptide whose amino-terminus shares similarities with its homolog, parathyroid hormone (PTH), and thus both can bind to the PTH 1 receptor (PTH1R). Although this protein is structurally and functionally similar to PTH, it was shown that PTHrP and PTH exert different effects on their target cells. For instance, in osteochondral development, PTH regulates calcium homeostasis, whereas PTHrP acts as a cytokine that modulates bone mass. Together, the two proteins regulate serum calcium levels and bone development [109]. Moreover, the targeted deletion of either PTH or PTHrP accelerates chondrocyte differentiation and induces skeletal malformations [110].

Studies have shown that PTHrP suppresses chondrocyte hypertrophy via both RUNX2-dependent and -independent mechanisms [111]. Several studies were conducted to explore the mechanism by which PTHrP inhibits chondrocyte hypertrophy. In a recent study, it was shown that PTHrP targets and activates HDAC4 and HDAC5 to suppress myocyte enhancer factor 2 (MEF2), which is required for the expression of RUNX2, a marker of chondrocyte hypertrophy [112]. The role of PTHrP in the chondrogenic differentiation of MSCs was also explored. In one study, over-expression of PTHrP in MSCs was found to upregulate TGF-β1 and the chondrogenic markers ACAN and SOX9, while downregulating the hypertrophic markers MMA13 and COL10. Additionally, it increased the GAG production in these MSCs. These results clearly suggest that the use of PTHrP, alone or in conjunction with MSC differentiation, could be a good and reliable treatment approach to preventing chondrocyte hypertrophy.

### 3.4. Inhibitors of Hypertrophic Markers

Although chondrocyte hypertrophy is generally treated via the induction of pro-chondrogenic signaling, it can also be prevented by inhibiting specific proteins, signaling molecules, or molecular markers of hypertrophy. Some known markers of chondrocyte hypertrophy include proteolytic enzymes, such as MMP13; hypertrophic markers, such as COL10, osteopontin, osteocalcin, and RUNX2; increased calcium deposition [6].

In a rat model of developmental dysplasia, it was determined that the early onset of cartilage degeneration is associated with increased MMP13 and COL10 expression, which is aggravated with age [113]. In another study, it was shown that deletion of the *MMP13* gene in mice decelerated OA progression, increased COL2 and proteoglycan expression, and decreased chondrocyte apoptosis. Moreover, the researchers also explored the effect of CL82198, a selective inhibitor of MMP13, on OA progression and found that it significantly decreased MMP13 activity both in vitro and in vivo [114]. Other selective inhibitors of MMP13, such as CGS-27023A, and their mechanisms are extensively discussed elsewhere [115]. Nevertheless, the inhibition of MMP13 has proven to be effective in treating OA progression by inhibiting early-onset chondrocyte hypertrophy.

Gene- and protein-based therapy is probably the most effective intervention for the prevention of or reduction in chondrocyte hypertrophy, as it directly involves the alteration of the expression of various genes at the molecular level. It offers an immediate response to the problem, and thus offers a faster solution for various diseases, including chondrocyte hypertrophy. However, since gene and protein therapeutics involve either the over-expression or down-regulation of genes, other downstream target genes might also be affected, and thus might produce side effects. Moreover, for better clinical applications, gene and protein interventions require targeted delivery, tissue-specific, and time-specific expression [116]. These problems, however, can now be solved by inventing gene and protein delivery systems that specifically target the injured site, while also promoting the consistent release or controlled expression of the target genes/proteins.

### 3.5. Small Molecules Preventing Chondrocyte Hypertrophy

As previously described, the chondrocyte hypertrophy has been announced to be related with various signaling pathways, such as Wnt [117], IHH [118,119], PTHrP [112,120], TGF-β [102], mitogen-activated protein (MAP) kinases [121,122].

Accordingly, many studies have tried to regulate these signaling pathways using small molecules. A study showed that the overexpression of an enhancer of zeste homolog 2 (EZH2) induces the Wnt/β-catenin signaling via histone methylation and causes chondrocytes to undergo hypertrophy and OA [123]. Another study found that the selective EZH2 inhibitor EPZ005687 decreases chondrocyte hypertrophy through Wnt/β-catenin signaling [124]. The Wnt signaling inhibitor SM04690 was shown to have therapeutic potential against OA and chondrocyte hypertrophy [125]. This compound downregulates the hypertrophic markers COL I and RUNX 2, and upregulates the chondrogenic markers SOX9, ACAN, COL2, and TGF-β1, and glycosaminoglycan content, in degenerated chondrocytes via the Wnt signaling pathway.

RCGD 423 is known as a small molecule, reported to reduce chondrocyte hypertrophy [126]. RCGD 423 was identified from screening 170,000 compounds to identify the ones that can modulate the glycoprotein 130 signaling. It was reported to reduce chondrocyte hypertrophy by suppressing the ERK and NF-κB pathways. A study reported that the p38 MAPK inhibitor SB303580 upregulates Bcl-2, which is downstream of the p38 MAPK signaling in the PTH pathway and downregulates the hypertrophic marker COL10 [121].

#### Chemical Interventions Found in Nature

In addition to the artificial chemical cues, the chemical cues present in nature were also assessed for their effects against chondrocyte hypertrophy. Curcumin has been reported to have anti-inflammatory and anti-tumor effects, and it inhibits chondrocyte hypertrophy through the IHH and Notch signaling pathways [127]. Cordycepin is a nucleoside adenosine, which has antioxidant and anti-inflammatory effects, and it has been reported that cordycepin inhibits chondrocyte hypertrophy though PI3K/Bapx1 and Notch signaling [128].

Hence, the traditional herbal medicine, Icariin has reported that icariin treatment significantly reduced the degeneration of OA cartilage and chondrocyte hypertrophy. Icariin downregulates IHH and hypertrophic markers, including MMP13 and COL10 [129]. Another study reported that a well-known flavonoid, vanillic acid, decreases chondrocyte hypertrophy and cartilage degeneration by regulating the MAPK and PI3K/AKT/NF-κB pathways [130]. Tauroursodeoxycholic acid (TUDCA) is known as one of the bile acids and a chemical chaperone. Arai et al. have observed that TUDCA not only increases the membrane fluidity and stability of TGF-β receptors in degenerated chondrocytes, but also upregulates chondrogenic markers, including SOX9 and COL2, and downregulates hypertrophic markers, such as RUNX2 and MMP13, in degenerated chondrocytes [131].

In summary, several small molecule candidates have been investigated for their therapeutic potential for chondrocyte hypertrophy. The therapeutic application of small molecules has various advantages, such as the possibility of large-scale production, simple modifications needed to engage the biological target and simple drug administration to patients. However, due to the lack of persistence, it is difficult to expect the therapeutic effect with single-dose administration. To overcome this hurdle, numerous researchers have investigated the development of drug delivery systems to enhance the sustainability of drug and long-term effects. Therefore, in the next section, three-dimensional environments for the advancement of drug delivery systems and prevention of chondrocyte hypertrophy will be discussed.

### 3.6. Three-Dimensional Environments Preventing Chondrocyte Hypertrophy

Given that cells are not arranged as monolayers (two-dimensional, 2D) in the human body, many researchers have sought to mimic the 3D architecture of tissues when culturing the corresponding cells. Thus, 3D-environment studies have been widely pursued due to the similarities between such in-vitro conditions and their in-vivo counterparts [132,133,134]. It has been shown that the 3D microenvironment can inhibit cell hypertrophy by modulating the cell morphology and niche [135,136]. In chondrocyte cultures, the 3D microenvironment inhibits hypertrophy and even converts hypertrophic chondrocytes to healthy chondrocytes [137]. Several biomaterials, such as silk fibroin, collagen, HA, and chondroitin sulfate (CS), are typically utilized to construct 3D microenvironments against chondrocyte hypertrophy. In this subsection, the characteristics of materials, their roles, and their current applications in preventing and treating chondrocyte hypertrophy, will be elaborated and discussed.

Fibroin is composed of three chains—light, heavy, and glycoprotein P25. These chains are connected to each other. The light and heavy chains are linked via disulfide bonds, while P25 is non-covalently linked via the heavy and light chains. Due to these characteristics, the silk fibroin scaffold has a porous structure, excellent mechanical property, and suitable degradation abilities for chondrogenic differentiation. A study found that chondrocyte hypertrophy is suppressed when chondrocytes are co-cultured with adipose-derived stem cells in a silk-fibroin hydrogel. This suppressive effect presumably results from reciprocal cross-talks, paracrine signaling, and the cell-niche regulation capacity of the silk-fibroin hydrogel [138] (Table 2).

Collagen is composed of amino acids that form a triple-helical structure, and is mostly found in fibrous tissues and the cartilage [139]. Among the different types of collagen, COL2 is the building block of articular cartilage and hyaline cartilage, formed of homogenous trimers of COL2A1. COL2 constitutes 50% of all the proteins and approximately 90% of the collagen in the articular cartilage. In one study, collagen hydrogel was found to induce cell proliferation and matrix production. Moreover, the chondrogenic genes ACAN, SOX9, and COL2 were upregulated, whereas the hypertrophic genes COL1 and COL10 were downregulated [140].

HA is an anionic non-sulfated glycosaminoglycan, widely distributed in the connective, epithelial, and nervous tissues. It is a polymer of disaccharides composed of D-glucuronic acid and N-acetyl-D-glucosamine, which are alternately linked via glycosidic bonds. The number of these repeating groups determines the molecular weight (MW) of HA, and various receptors, such as CD 44, bind to HA, depending on the MW of HA. For example, clustered CD44 can bind to the high-MW HA, whereas various receptors, such as CD44, a receptor for HA-mediated motility (RHAMM), and intercellular adhesion molecule-1 (ICAM-1), bind to the low-MW HA [141]. As described above, cells typically have three types of surface receptor that can bind to HA, which are CD44, RHAMM, and ICAM-1 [142]. These receptors are cell-adhesion molecules and can also be combined with other surface receptors. CD44 interacts with the TGF-β receptor 1 (TGFBR1) and recruits TGF-β receptor 2 (TGFBR2), which is involved in the chondrogenic signaling pathway. Furthermore, CD44 mediates cell aggregation, proliferation, migration, and differentiation as a result of its interaction with HA. ICAM-1 binds to integrin αMβ2, leukocyte function associated antigen-1 (LFA-1), and fibrinogen. The interaction of HA with ICAM-1 enhances ICAM-1, downregulates pro-inflammatory cytokines, decreases chondrocyte hypertrophy, and increases cartilage regeneration [143,144]. Additionally, several studies found that mixing collagen with HA has a synergistic effect on enhancing the differentiation of chondrocytes and suppressing chondrocyte hypertrophy [145,146].

CS, one of the building blocks of the cartilage, has also been proven to have an inhibitory effect on chondrocyte hypertrophy. CS is a sulfated GAG composed of a chain of alternating sugars, which are abundant in hyaline cartilage. Since CS provides resistance to compression, it is widely used in the regeneration of the cartilage tissue [147]. PEG/CS hydrogel systems have been shown to increase the expression of chondrogenic markers and the secretion of the ECM components, while decreasing the expression of the hypertrophy marker COL10 [148,149].

In addition to the above-mentioned factors, cytokines are also used in conjunction with 3D microenvironments to inhibit chondrocyte hypertrophy. In one study, a 3D culture of chondrocytes with human recombinant FGF18 strongly downregulated COL1 and upregulated SOX9, concurrently stimulating the proliferation of chondrocytes [150]. Another study showed that chondrocyte hypertrophy was inhibited on a scaffold composed of matrilin-3 and poly-L-lactic acid (PLLA), compared with the cells on the PLLA-only scaffold [151].

Meanwhile, the scaffold derived from human tissue was also studied for chondrocyte hypertrophy treatment. Several studies showed that the human cartilage matrix induces the chondrogenic differentiation of adipose-derived mesenchymal stem cells (A-MSCs) and enhances ECM accumulation [152,153]. These studies also showed that COL2 and SOX9 are upregulated, whereas the hypertrophic marker COL1 is downregulated, under these conditions. In another study, it was determined that the cartilage matrix can upregulate COL2 and suppress chondrocyte hypertrophy. Similarly, another study using the chondrocyte matrix observed that osteocalcin, COL1, and COL10 were downregulated during the progress of chondrocytes hypertrophy [154].

Recently, decellularized ECM has been utilized in 3D studies to inhibit chondrocyte hypertrophy. Several studies showed that the decellularized ECM of BM-MSCs or synovium-derived mesenchymal stem cells (S-MSCs) downregulates COL10A1, ALP and MMP13 during the chondrogenic differentiation of MSCs [155,156].

In conclusion, polymer-based or cytokine-loaded 3D scaffolds, and decellularized ECM derived from tissues and cells, each have their advantages in inhibiting hypertrophy. A combination of these materials has also been proven to have a synergistic effect on preventing hypertrophy.

In sum, the three-dimensional environment provides a body-mimicking environment for chondrocytes, and which is effective in the transmission of biophysical signal. In addition, the three-dimensional environment makes it easy to load the growth factor or chemicals that have a therapeutic effect on OA. Therefore, the 3D environment is able to provide complex signals of biochemical and physical signals for the treatment of chondrocyte-hypertrophy-induced OA. However, it still has many hurdles (immune response that occurs when transplanted into the body, cytotoxicity of the biomaterials, etc.) to overcome before its clinical application. Therefore, new strategies to overcome these hurdles must be investigated. In the next section, the exosomes and how exosome-based therapies can be utilized for the prevention of chondrocyte hypertrophy will be discussed.

### 3.7. Exosomes

Exosomes are small vesicles made of a phospholipid bilayer, produced and released by various eukaryotic cells. These vesicles form one of the three large groups of extracellular vesicles (EVs), with the other two being apoptotic bodies and micro vesicles. Exosomes range from 30 nm to 100 nm in diameter and play crucial roles in various biological processes, including angiogenesis, immune modulation, cell–cell communication, and maintenance of tissue microenvironments. Exosomes regulate these processes by acting as molecular cargos that deliver their components, such as microRNAs (miRNAs), mRNAs, DNAs, proteins, lipids, and cellular by-products, from one cell to another cell, or to the microenvironment of the originating cell [157,158,159]. Due to these characteristics, exosomes have been a point of interest due to their therapeutic potential for various diseases, including OA. Exosomes can work as negative or positive regulators of cartilage development. In this sub-section, the different aspects of exosomes as a potential modality of OA treatment will be described.

### 3.8. Stem Cell-Derived Exosomes

One current strategy for treating OA is the application of exosomes derived from stem cells, such as umbilical-cord-derived MSCs (U-MSCs), A-MSCs, S-MSCs, BM-MSCs, and iPSCs [160,161,162,163]. In fact, the use of exosomes from these cells is one of the strongest candidates to approach for the treatment of OA. In one study, transplanted MSCs suppressed OA; however, this effect was not because of their engraftment or differentiation into other cell types, but possibly due to the factors they secrete [164]. As the authors suggested, such factors may have induced the tissue regeneration [165], and thus MSCs may have paracrine effects, enhancing regeneration and suppressing inflammation. Meanwhile, another study reported that MSC-derived exosomes prevent cartilage degeneration by up-regulating chondrogenic markers (COL2 and ACAN) and downregulating hypertrophic markers (MMP 13 and ADAMTS5), and the inflammation-marker-inducible nitric oxide synthase (iNOS) [166]. Moreover, a similar study reported that human embryonic MSC-derived exosomes promote osteochondral regeneration via increases in GAG and COL2 expression [167]. The same study also assessed the effect of MSC exosomes in temporomandibular joint osteoarthritis (TMJOA) and observed that these exosomes attenuated the inflammation and restored the matrix homeostasis by downregulating MMP13 and IL-1β and suppressing GAG synthesis [168]. Additionally, A-MSC-derived exosomes enhance the polarization of macrophages into the M2 phenotype, which has anti-inflammation properties [169]. Another study reported the therapeutic capacity of iPSC-derived MSC exosomes (iMSC-EXOs). In that study, it was determined that iMSC-EXOs have a stronger therapeutic effect on OA by stimulating the proliferation and migration of chondrocytes compared with synovial membrane MSCs [170]. Although the mechanisms by which stem cell-derived exosomes regulate OA are not yet fully understood, the current evidence indicates that these exosomes suppress the indications of OA, offering potential approaches for the treatment of OA.

### 3.9. Exosomes Derived from Other Cell Types

**Figure 2:**
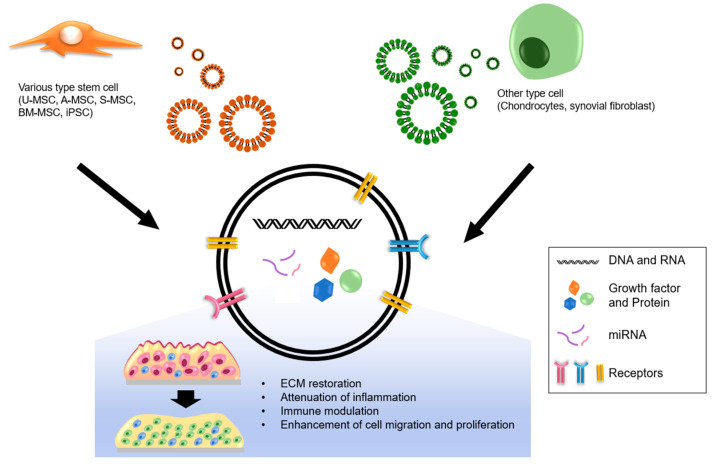
The exosomes and their therapeutic effects on OA.

Although many studies have focused on the use of stem-cell-derived exosomes and their therapeutic effects, other cell types also secrete exosomes that may be of use for OA. Exosomes derived from chondrocytes or synovial fibroblasts have recently been explored as possible modalities of OA treatment [171]. For instance, it has been shown that exosomes secreted by the resident chondrocytes of the cartilage tissue are one of the key factors affecting cartilage physiology [6]. It has been found that exosomes derived from osteoarthritic chondrocytes are enriched in 22 miRNAs and depleted in 29 miRNAs, compared with the cellular levels. Further, miR-95-5p has been found to attenuate OA progression by inhibiting chondrocyte hypertrophy [172]. The same authors also found that miR-95-5p is overrepresented in exosomes secreted by hMSC-derived chondrocytes, and the overexpression of miR-95-5p suppressed OA in mouse models by inhibiting the Wnt5a signaling, which is a pro-hypertrophic pathway [173]. Meanwhile, a study found that exosomes derived from synovial fibroblasts modulate the macrophage secretum [171]. However, several studies suggested that these exosomes exert negative effects on articular chondrocytes by upregulating MMP13 and ADAMTS5 and downregulating ACAN and COL2 [174]. Thus, it appears that the roles of these exosomes in chondrocyte physiology are not yet fully understood. In a comparative study, it was reported that the EVs of the synovial fibroblasts derived from the synovial fluid of OA patients differ depending on gender. According to the same study, the levels of the miRNAs in these EVs and their cellular levels are also different between the two genders [175]. Therefore, further studies are needed to clarify the functions of exosomes derived from synovial fibroblasts as well as to determine the effects and therapeutic potentials of exosomes derived from stem cells and other cell types (Figure 2).

In summarize, the therapeutic application of exosomes has great potential for the treatment of hypertrophy-induced OA due to its biocompatibility, low or no immunogenicity, and multiple synergistic effects (miRNAs, mRNAs, proteins, and growth factors, etc.). However, the mechanism and controversial points between each study must be investigated to advance the therapeutic applications of exosome.

## 4. Conclusions and Future Perspectives

**Figure 3:**
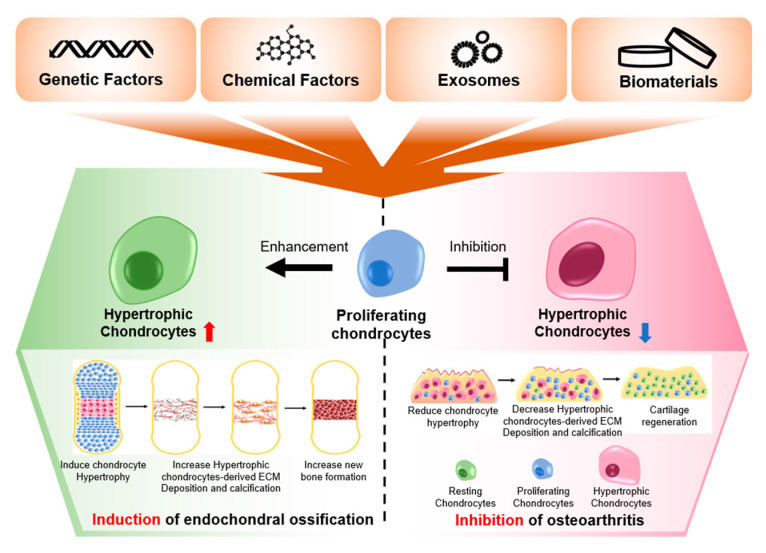
The expected effects of preventing or promoting chondrocyte hypertrophy, using various factors.

The current society is getting older, and thus the rates of various types of aging-associated diseases are also increasing. One of these chronic diseases, OA, is not only a representative aging-associated disease, but also a degenerative disease, with low levels of self-regeneration. To prevent or treat this disease, many scientific studies and clinical trials have been conducted, but the pathology of this disease and an effective treatment modality have yet to be determined. This review suggest that this shortcoming is due to the lack of understanding of chondrocytes and their niche in the cartilage. Therefore, to find the ultimate treatment for OA, a deeper understanding of chondrocytes is essential. Since chondrocyte hypertrophy is the primary symptom present in both the early and late stages of OA, it is essential to delve into this cellular process further. According to current knowledge, the dual mechanism of chondrocyte hypertrophy in bone development and OA progression was demonstrated in this review. Chondrocyte-hypertrophy-related genes, such as MMPs, ADAMTS, RUNX2, COL10, C/EBPβ, HIF-2α, ALP, and OPG, were increased, and chondrogenic genes such as COL2 and SOX9 were decreased in chondrocyte when hypertrophy occurred. The alternative gene expression caused by chondrocyte hypertrophy led to destruction and reduced functioning of cartilage tissue during the progression of OA and generated and maintained the bone tissue during endochondral ossification in the developmental stage. Additionally, this review has listed, elucidated, and discussed the current strategies (genes, biomolecules, small molecules, 3D microenvironments, and exosomes) for treating the OA induced by chondrocyte hypertrophy (Figure 3).

These factors may be used to modulate chondrocyte hypertrophy according to the needs of endochondral ossification and osteoarthritis. Administration of the factors that induce hypertrophy is not suitable for OA therapy, because this will promote the progression of OA, but it is useful for bone tissue regeneration, by promoting endochondral ossification. For example, it has been reported that the chondrocyte hypertrophy-related gene, MMP13, which is known to lead to the destruction of cartilage tissue, promotes bone regeneration [176]. In addition, the administration of SPRY4 is inappropriate for OA therapy because it causes chondrocyte hypertrophy, which hinders cartilage regeneration; however, it is appropriate for bone regeneration because chondrocyte hypertrophy is involved in endochondral ossification for bone regeneration [177]. However, the administration of inhibitors for chondrocyte hypertrophy-related genes such as siRNA may suppress bone regeneration. Since the regulation of chondrocyte hypertrophy can lead to the opposite results, depending on the means of application, an accurate understanding of the dual function of chondrocyte hypertrophy in OA and endochondral ossification is required.

In conclusion, this review detailed the dual function of chondrocyte hypertrophy and the current strategies for treating OA. This review suggests that chondrocyte hypertrophy is essential in the maintenance of osteochondral tissues; however, proper regulation of chondrocyte hypertrophy is needed to prevent and control hypertrophy-induced OA. If the various factors in the regulation of chondrocyte hypertrophy shown in this review are properly used, researchers can induce synergistic effects in OA treatment as well as bone regeneration treatment through the integration of treatment strategies. These possibilities can be realized by conducting further research on the underlying mechanisms of hypertrophy-induced OA and their relevant clinical applications.

## Figures and Tables

**Table 1:** Summary of gene and protein interventions for chondrocyte hypertrophy.

Type	Factors	Properties	In-Vitro Results	In-Vivo Results	Reference
Genetic Intervention	SOX9	Master regulator of Chondrogenesis	Adenoviral transduction improves chondrogenesis, over-expression enhances BMMSC chondrogenesis	SOX9 promotes healthy growth plates and articular cartilage	[74]
	Histone Deacetylase 4 (HDAC4)	Transcription factor, epigenetic regulator of histone proteins	Adenoviral transduction in rat chondrocyte prevents cartilage degeneration, interacts with and inhibits RUNX2 and MEF2C; Over-expression promotes TGF-B1 expression	Over-expression inhibits chondrocyte hypertrophy similar to RUNX2-loss of function phenotype	[89,90]
	NKX3.2	Transcription factor, human homolog of the bagpipe gene (BAPX1)	Retroviral transduction prevents chondrocyte maturation and hypertrophy; over-expression enhanced COL2A1 and SOX9 expression	Over-expression causes skeletal dwarfism by delaying cartilage hypertrophy	[96]
	E2F1	Transcription factor	E2F1 expression delays early and late phase differentiation of ATDC5 cells	over-expression prevents chondrocyte maturation indicated by reduced hypertrophic zone and disorganized growth plate	[97]
Protein Intervention	TGF-Beta	Growth factor involved in chondrocyte and cartilage development	exogenous TGFB1 prevents terminal differentiation of chondrocytes; TGFB3 enhanced chondrogenesis and prevents hypertrophy in ASC	TGFB1 activation inhibits BMP signaling in cartilage	[103,106]
	PTHrP	Homolog of the parathyroid hormone	Activates HDAC4/HDAC5 suppressing MEF2 and RUNX2 that promotes hypertrophy; over-expression reduces hypertrophic markers MMP13 and COL10 in MSC	Expression of PTHrP in IHH mutant mice prevents hypertrophy of chondrocyte	[6]

**Table 2:** Various polymer- or bio-derived scaffolds propose for use in the treatment of OA.

Type	Classification		Characteristics and Function	Reference
Polymer-derived scaffold	Biomaterials (Natural polymer)	Silk fibroin	Composed of three chains (light, heavy, and glycoprotein P25) Upregulates chondrogenic proteins (SOX9, COL2, and ACAN) Downregulates hypertrophic markers (MMP-13 and COL10)	[138]
		Collagen	Composed of amino acids that form a triple-helix structure. Mostly found in the cartilage and fibrous tissues. Stimulates cell proliferation and matrix production, upregulates chondrogenic proteins (ACAN, SOX9, and COL2) Downregulates hypertrophic markers (COL1 and COL10)	[139,140]
		Hyaluronic acid	Composed of D-glucuronic acid and N-acetyl-D-glucosamine that are linked via alternating glycosidic bonds. Has three main receptors (CD44, ICAM-1, and RHAMM). Induces ICAM-1 mediated inflammatory activation and suppresses chondrocyte hypertrophy and matrix calcification. Upregulates cartilage-related signals (COL2 and ACAN) Downregulates ALP, COL2, and MMP13	[141,142,143,144,145,146]
		Chondroitin sulfate	Sulfated GAG Composed of chain of alternating sugars. Upregulates ACAN and COL2 Downregulates COL10	[147,148,149]
Bio-derived scaffold	Tissue	Articular cartilage	Hyaline cartilage covering the articular surfaces of bones Stimulates ECM accumulation and upregulates proteoglycans, COL2, and GAG Downregulates COL10	[152,153]
	Cell	Chondrocyte	Embedded in the cartilage. Secretes cartilage ECM. Upregulates chondrogenic markers (SOX9. COL2, ACAN, and GAGs) Downregulates hypertrophic markers (Osteocalcin, COL1, and COL10)	[154]
		Marrow- or synovium-derived stem cell	Mesenchymal stem cells that can differentiate into chondrocytes Upregulates COL2, ACAN, and SOX9 for chondrogenesis Downregulates COL10, ALP, and MMP 13	[155,156]
